# Coronary artery size and origin imaging in children: a comparative study of MRI and trans-thoracic echocardiography

**DOI:** 10.1186/s12880-015-0095-7

**Published:** 2015-10-27

**Authors:** Tarique Hussain, Sujeev Mathur, Sarah A. Peel, Israel Valverde, Karolina Bilska, Markus Henningsson, Rene M. Botnar, John Simpson, Gerald F. Greil

**Affiliations:** Division of Imaging Sciences, King’s College London, NIHR Biomedical Research Centre at Guy’s & St Thomas’ NHS Foundation Trust, London, UK; Department of Paediatric Cardiology, Evelina London Children’s Hospital, Guy’s & St Thomas’ NHS Foundation Trust, Westminster Bridge Road, London, UK; Department of Pediatrics, UT Southwestern Medical Center, Children’s Medical Center, 1935 Medical District Drive, Dallas, TX USA

**Keywords:** Child, Infant, Newborn, Adolescent, Magnetic resonance imaging, Echocardiography, Coronary vessels

## Abstract

**Background:**

The purpose of this study was to see how coronary magnetic resonance angiography (CMRA) compared to echocardiography for the detection of coronary artery origins and to compare CMRA measurements for coronary dimensions in children with published echocardiographic reference values.

**Methods:**

Enrolled patients underwent dual cardiac phase CMRA and echocardiography under the same anesthetic. Echocardiographic measurements of the right coronary artery (RCA), left anterior descending (LAD) and left main (LM) were made. CMRA dimensions were assessed manually at the same points as the echocardiographic measurements. The number of proximal LAD branches imaged was also recorded in order to give an estimate of distal coronary tree visualization.

**Results:**

Fifty patients (24 boys, mean age 4.0 years (range 18 days to 18 years)) underwent dual-phase CMRA. Coronary origins were identified in 47/50 cases for CMRA (remaining 3 were infants aged 3, 9 and 11 months). In comparison, origins were identified in 41/50 cases for echo (remaining were all older children).

CMRA performed better than echocardiography in terms of distal visualization of the coronary tree (median 1 LAD branch vs. median 0; *p* = 0.001).

Bland-Altman plots show poor agreement between echocardiography and CMRA for coronary measurements. CMRA measurements did vary according to cardiac phase (systolic mean 1.90, s.d. 0.05 mm vs. diastolic mean 1.84, s.d. 0.05 mm; *p* = 0.002).

**Conclusions:**

Dual-phase CMRA has an excellent (94 %) success rate for the detection of coronary origins in children. Newborn infants remain challenging and echocardiography remains the accepted imaging modality for this age group. Echocardiographic reference ranges are not applicable to CMRA measurements as agreement was poor between modalities. Future coronary reference values, using any imaging modality, should quote the phase in which it was measured.

## Background

In infants, it is appreciated that currently, echocardiography is the first line imaging method for delineation of the origin and course of the proximal portion of the coronary arteries [1]. However, echocardiographic imaging becomes progressively more difficult in children and adolescents due to patient size and poor transthoracic ultrasound windows.

Normal values exist for coronary dimensions using echocardiography, [2, 3] but no such references exist for coronary magnetic resonance angiography (CMRA). The difficulty exists in producing normal MRI data is that young children and infants would require sedation or general anesthesia. This information, however, may be gained by cross-referencing MRI and echo data, so that MR dimensions can be validated systematically against echo-derived dimensions. This will be important for clinical settings, such as Kawasaki disease (or coronary allograft vasculopathy), where MRI is used for longitudinal follow up of coronary arteries.

In the setting of aneurysmal coronary segments and larger coronary arteries (>3 mm in adults), it has already been shown that CMRA is very accurate [4]. The purpose here is to be able to develop a reference for coronary dimensions in children to identify whether the segment in question is indeed dilated. This is particularly important for longitudinal follow-up and when echocardiographic windows are poor.

## Methods

Institutional Review Board approval was obtained for this study (St. Thomas’ research ethics committee reference 07/Q0704/3). The inclusion criterion in this prospective study was any patient undergoing a clinical cardiovascular MRI with 3d-whole heart acquisition under general anesthesia at our institution. Written, informed consent was obtained in each case from the patient (if older than 16 years) or from the legal guardian (if patient was younger). Enrolled patients underwent dual phase whole-heart 3D balanced steady state free precession (b-SSFP) imaging and echocardiography under general anesthesia [5].

### MRI

Cardiac MRI was performed using a 1.5 Tesla Achieva clinical MR scanner (Philips Healthcare, Best, NL). The 3d whole heart approach, described by Beerbaum et al. [6] was implemented with noticeable changes. It has been recently demonstrated that dual-phase imaging (end-systole and mid-diastole) can improve coronary imaging by providing the ability to retrospectively select the optimum phase to be used for analysis [7]. Hence this approach was used for this study.

As with standard 3D SSFP sequences, the dual-phase sequence is respiratory-gated and ECG-triggered with a fat saturation pre-pulse to null fat signal and a T_2_ pre-pulse to improve the myocardium to blood pool contrast [8]. Imaging was acquired in a sagittal orientation (repetition time (TR) /echo time (TE) = 3.4/1.7 ms, flip angle 90°, 60–120 slices, isotropic resolution of 1–1.5 mm^3^, reconstructed resolution 0.5–0.75 mm^3^, acquisition window of 40–75 ms). For children 15 kg or less, a two-element coil was used (Flex M or Flex S). For children over 15 kg, a five-element cardiac coil was used. Parallel imaging with a SENSitivity Encoding (SENSE) acceleration factor of 2 in the antero-posterior direction was also used [9]. Trigger delays were set for end-systole and mid-diastole. The cardiac rest periods were assessed with a high temporal resolution, balanced steady-state free precession (SSFP), four-chamber cine (TR = 2.8–3.6 ms, TE = 1.4–1.8 ms, no. of lines acquired per heart beat = 3 to 20, flip angle 60°, 6-mm-thick sections, 240–300 mm field of view, 60–80 phases). These parameters were adjusted accordingly to maintain temporal resolution with minimal interpolation. The dual-phase sequence employs navigators for each cardiac phase with a respiratory gating window of 3 mm. Data is only accepted if both navigators, in any given cardiac cycle, fall within this gating window.

In addition, it has been shown that an automated programme is capable of more accurate definitions of cardiac rest periods than visual inspection [10]. It was therefore hoped that clearer definitions for cardiac rest-periods would produce a similar effect. The mid-diastolic period was taken from cessation of movement of RCA (i.e., pause in visible filling of RV) to the beginning of atrial systole. This stringent definition covers both RCA and LAD diastolic rest periods [11]. The end-systolic period was taken from cessation of movement of the RCA (corresponding to lowest RV volume) to just before the beginning of opening of the tricuspid valve. The acquisition window was set according to the shortest rest period. Coronary dimensions were assessed manually at the same points as the echocardiographic measurements. The number of proximal LAD branches imaged was also recorded in order to give an estimate of distal coronary tree visualization.

Coronary origins were classified as abnormal if both echo & MRI observers agreed, without doubt, that they were abnormal. In the case where modalities disagreed, reference was made either to earlier diagnostic catheterization or to previous surgical notes.

### Echocardiography

Comprehensive echocardiographic studies were performed using the Philips IE33 ultrasound system (Philips Inc., Andover, MA, USA). 2-Dimensional echocardiography was performed using age appropriate probes (S5-1, S8-3, S12-4). The highest frequency probe yielding adequate depth penetration was employed. The Washington protocol for coronary dimensions was followed [3]. This protocol was chosen in preference as the measurement process is clearly defined. Images of the proximal coronary arteries (right coronary artery (RCA), left mainstem (LM) and left anterior descending (LAD)) were recorded as digital cines using short axis (in preference but alternatively, modified superiorly-tilted long-axis view for LAD was used). Operator assessment of coronary origins was recorded and stored cines were used to assess coronary dimensions.

Measurements of the RCA, LAD and LM were made from inner edge to inner edge, excluding points of branching [12]. LM was measured at its mid-point and the LAD/ RCA was measured 0.2 to 0.5 mm from its origin [3]. Measurements and imaging were performed by three experienced operators (SM, JS and KB, all with >5 years experience in coronary echocardiography in congenital heart disease).

Current echocardiographic data does not specify the point of the cardiac cycle (systole or diastole) over which measurements should be taken [3]. In addition, practically, it is often difficult to get images in both phases. Therefore, only one measurement was taken for each coronary measurement but it was also recorded as to which phase the measurement was taken, in order to compare it fairly to MRI. The number of proximal LAD branches imaged was also recorded in order to give an estimate of distal coronary tree visualization.

#### Statistical methods

First, bland-Altman plots were used to assess agreement between MRI and echocardiography for coronary dimensions. Using information gleaned from this analysis, regression analyses were planned in order to describe the relationship between MRI and echocardiographic measurements.

Distal coronary visualization, assessed by number of LAD branches visualized, was compared between modalities using Wilcoxon Signed Ranks test.

A repeated measures analysis of variance models for coronary dimensions was constructed in order to assess if the coronary distensibility in children results in different dimensions according to cardiac phase. A repeated measures model was used in order to account for within-subject and between-subject variability arising from testing multiple segments over two phases in each patient. Assumptions of sphericity were formally tested, and accepted, using Mauchly’s test. The Bonferroni correction is used to correct for multiple comparisons.

Statistical analyses were performed on SPSS (version 19) 2010. Variables are described using mean ± standard deviation (s.d.).

## Results

### Subjects

Fifty children participated in this study prospectively with a mean age of 4 ± 4.4 years (range 18 days to 18 years). This included 24 boys (mean age 4.5 ± 4.6 years) and 26 girls (mean age 3.6 ± 4.3 years; no significant age difference according to sex, *p* = 0.46 by independent samples *t*-test). Mean weight was 15.5 ± 11.6 kg (range 3.7 to 52 kg) and mean height was 94 ± 29 cm. 48 children underwent imaging for follow-up of congenital heart disease, 1 child for cardiomyopathy and 1 for resolved Kawasaki disease.

### Reference values

**Fig. 1:**
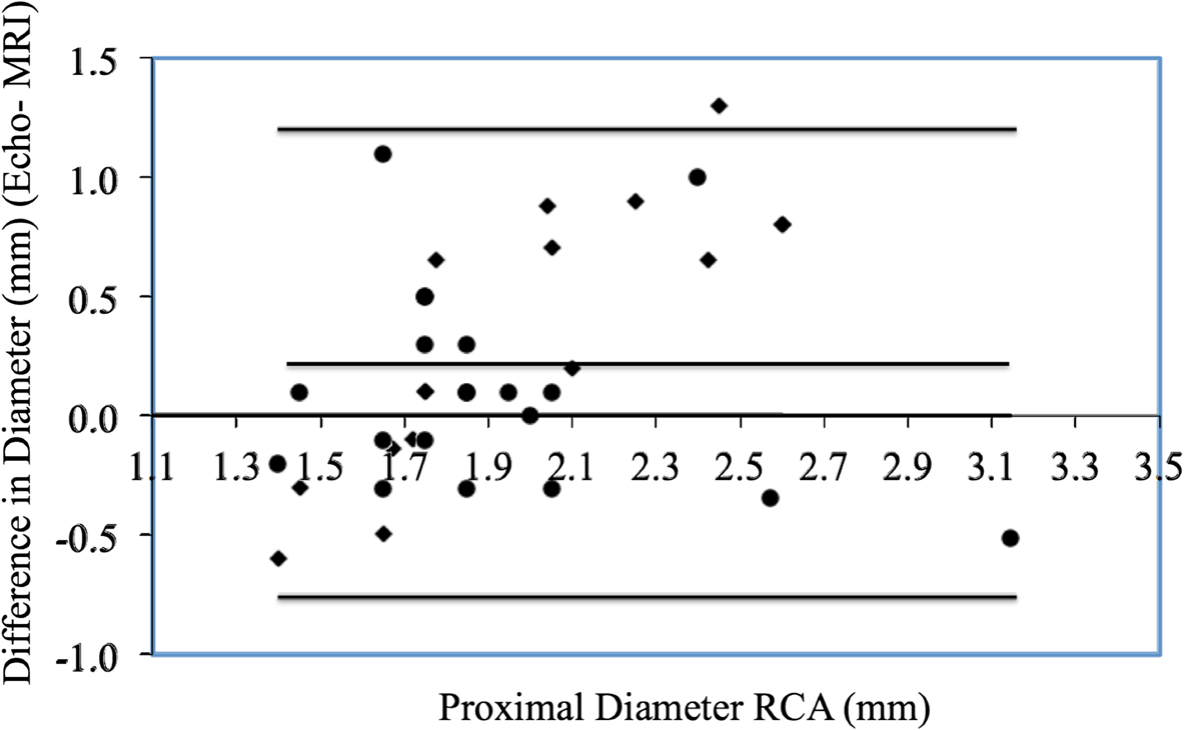
Bland Altman Mean vs. Difference plots for Echo and MRI RCA measurements. ● Diastolic measurements. ♦ Systolic measurements

**Fig. 2:**
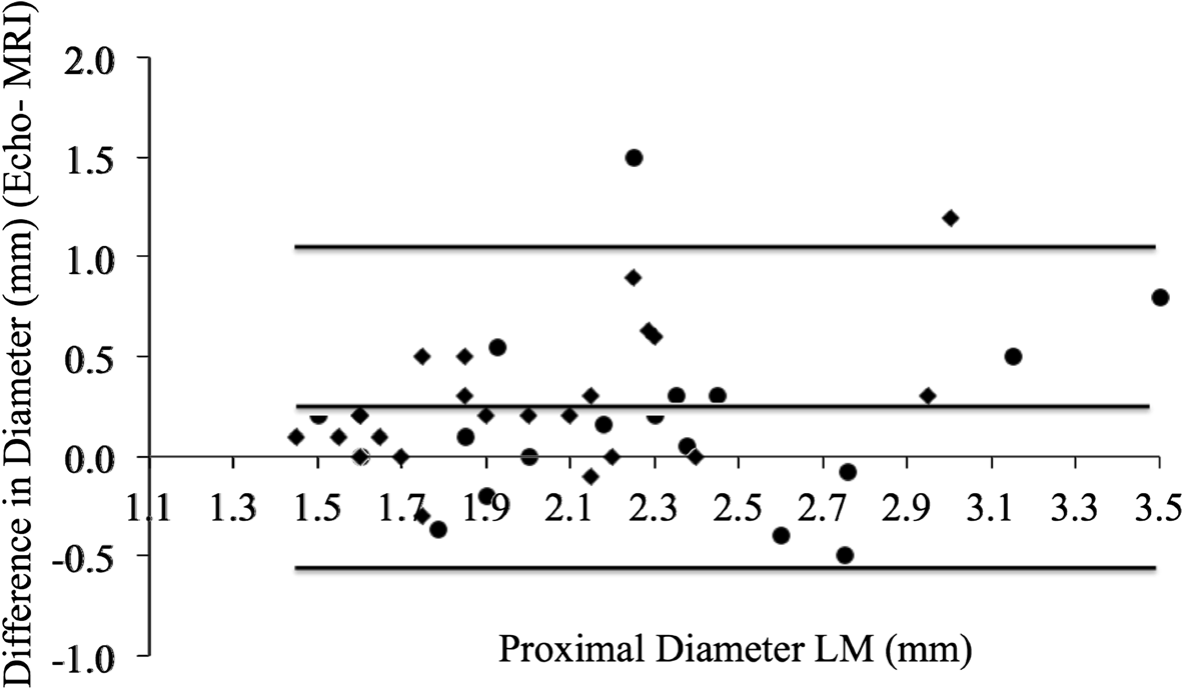
Bland Altman Mean vs. Difference plots for Echo and MRI LM measurements. ● Diastolic measurements. ♦ Systolic measurements

**Fig. 3:**
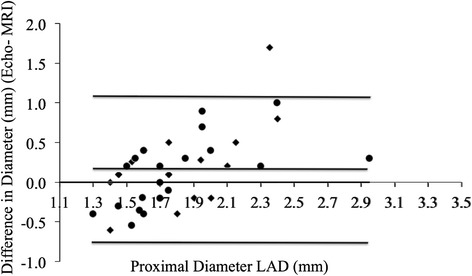
Bland Altman Mean vs. Difference plots for Echo and MRI LAD measurements. ● Diastolic measurements. ♦ Systolic measurements

Bland-Altman plots (Figs. 1, 2 and 3) show a systematic bias between echocardiographic and MRI measurements for all coronary measurements (i.e., as the coronary artery gets larger, the greater the discrepancy between echocardiography and MRI).

**Fig. 4:**
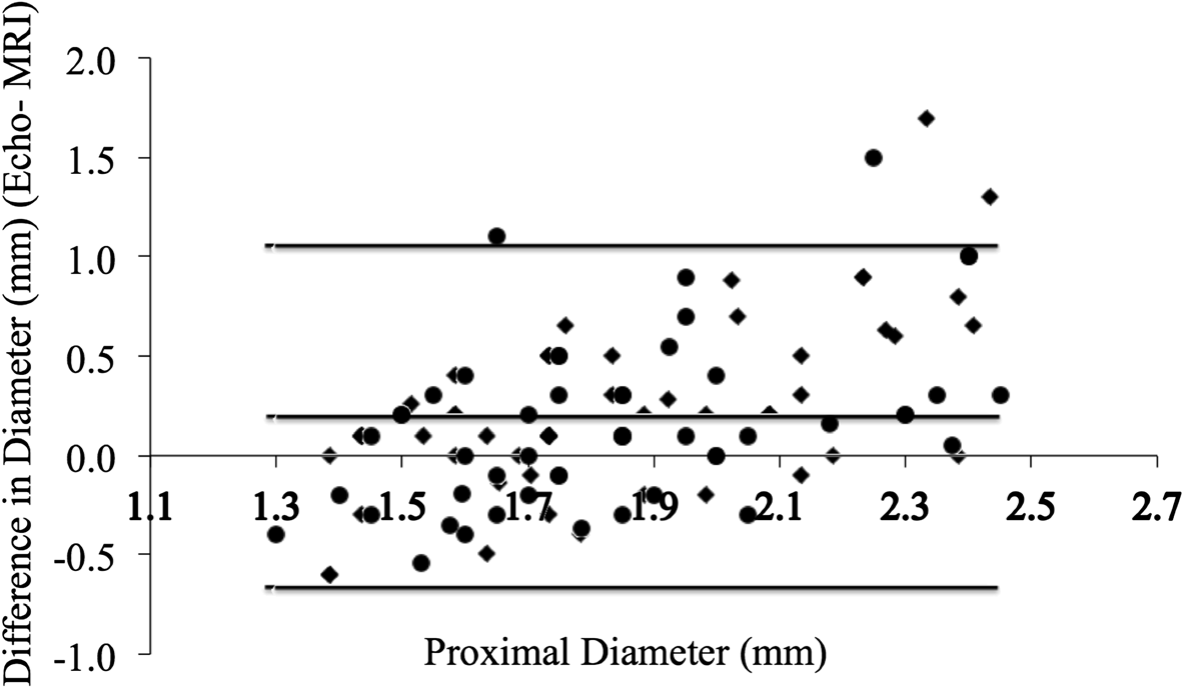
Bland Altman Mean vs. Difference plots for all Echo and MRI measurements with outliers excluded. ● Diastolic measurements. ♦ Systolic measurements

However, this may be more usefully summarized by ignoring the segment imaged, removing outliers and pooling data. If this is done we get a simple message: there is indeed a systematic bias for CMRA dimensions between 1 and 2.5 mm. This is demonstrated on the Bland-Altman plot (Fig. 4). More importantly, the accuracy between echo and MRI measurements is not clinically acceptable. The scatter clearly shows that, at dimensions between 1.3 to 2.5 mm, the spread of agreement is more than 0.5 mm at all points. Hence no further analysis was made to describe the correlation (i.e., regression analysis was not felt appropriate due to poor agreement).

### CMRA evaluation

Operator certainty regarding the origin of the three main coronary arteries (RCA, LAD and left circumflex (LCx)) was achieved in 47 out of 50 cases for MRI. The three uncertain cases were infant below a year of age (3, 9 and 11 months) and with heart rates >100 bpm. In comparison, certainty was achieved in 41 out of 50 cases for echo. The nine uncertain cases were older patients (mean 4.8 years, range 9 months to 18 years). In two cases, MRI detected abnormalities in coronary origin classified as normal with certainty on echocardiography. These two cases had confirmed abnormalities on surgery. Both were slightly older children (13 months and 8 years age respectively). The 13-month-old child had an anomalous origin of the left circumflex origin from the right coronary artery. The 8-year old child had ventriculo-arterial discordance and had a single coronary artery from left-hand sinus two (Fig. 6).

**Fig. 5:**
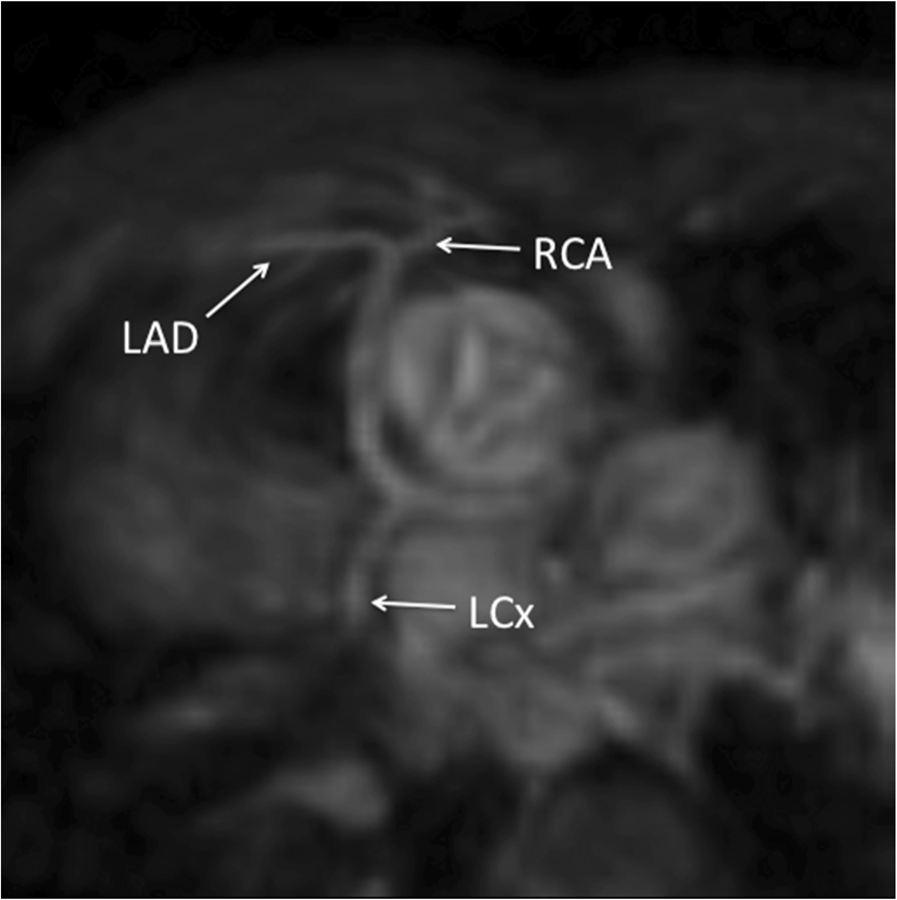
3-year old girl with Dextrocardia and Tricuspid Atresia. Single Coronary artery imaged with MRA in systole. Echocardiographic data corresponded with MRA findings

**Fig. 6:**
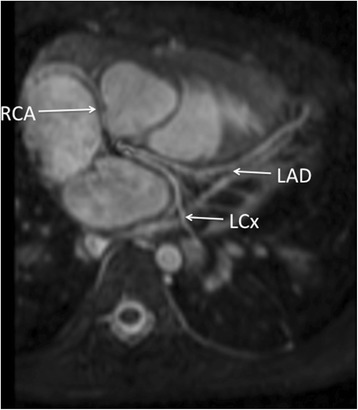
8-year old boy with ventriculo-arterial discordance showing single coronary artery. Echocardiography was not able to demonstrate this

**Fig. 7:**
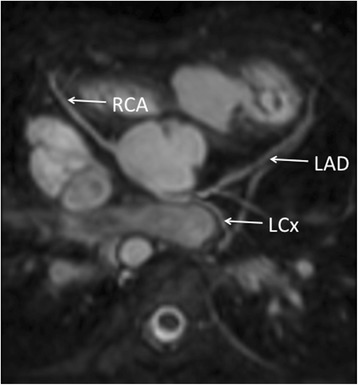
7-year old girl with repaired pulmonary atresia and ventricular septal defect. RCA arises from posterior non-coronary cusp. Findings were confirmed by echocardiography

In total, there were 6 abnormal coronary origins. 5 out of 6 were identified by MRI and the remaining one was classified as unsure. 4 out of 6 were correctly identified on echocardiogram with the remaining two, incorrectly classified as normal as described above. (Sample images: Figs. 5, 6 and 7).

Overall, CMRA performed better than echocardiography in terms of distal visualization of the coronary tree, as defined by number of proximal LAD branches imaged (*p* = 0.001 by Wilcoxon Signed Ranks test). The median number of LAD branches on MRI was 1 (interquartile range 0 to 2) and on echo was 0 (interquartile range 0 to 1).

CMRA measurements did vary according to cardiac phase. The repeated measures model demonstrates that the systolic coronary dimension (estimated marginal mean 1.90 ± 0.05 mm) is greater than the diastolic measurement (estimated marginal mean 1.84 ± 0.05 mm (*p* = 0.002).

## Discussion

This study has important implications for the follow-up of Kawasaki disease in particular. MRI is particularly useful for follow-up of Kawasaki Disease and Coronary Allograft Vasculopathy as anatomic evaluation can be combined with functional evaluation with pharmacological stress testing. Intra- and inter-observer error in coronary measurements for both echocardiography and CMRA have been previously separately investigated and shown to be low for both modalities [3, 7]. However, this study shows poor agreement between techniques. This may be in part, due to the current difficulty achieving sub-millimeter acquired resolution for coronary MRA in small children. The study resolution of 1–1.5 mm^3^ acquired (0.5–0.75 mm^3^ reconstructed) was chosen in order to give the optimum image quality as further increase in resolution would cause reduction in signal-to-noise ratio and would result in sub-optimal image quality. Improvements in image quality in infants should be the subject of future studies. It is hoped that, in the future, specific normative coronary dimensions for CMRA are available. The difficulty imaging normal volunteers in this age group has precluded this to date. Hence, until such data becomes available, the utility of coronary measurement below 3 mm lies in serial follow-up rather than for normative scoring. In particular, when reporting MRI coronary dimensions, we should not use published z-score values for echocardiography [3]. In the past, clinicians have merely assumed that dimensions were normal on CMRA if they equated to normal published echocardiographic z-scores. This study shows that this assumption is erroneous. One may argue that this result was to be expected because echocardiography relies on high contrast in the vessel wall and low contrast in the lumen, whereas CMRA is the opposite. Although ‘black-blood’ coronary MRI exists [13] and contrast-enhanced echocardiography exists, the purpose of this study was to evaluate the commonly used clinical echocardiographic and MRI techniques.

In keeping with previous studies, this study shows an excellent (94 %) success rate for CMRA detection of coronary origins in children [7, 14, 15]. Dual-phase imaging may be advantageous as it has been previously shown that image quality of each proximal coronary segment can vary unpredictably within the same patient according to selection of the systolic or diastolic rest period for imaging [7]. Infants with high heart rates remain challenging [14] and echocardiography remains the modality of choice for this age group. Naturally, echocardiography remains the first choice for cardiac imaging in all children due to the ease of application and the acceptability from a child’s perspective. Success rates are difficult to report from literature, as the technique is operator dependent with some operators showing excellent accuracy [16–18]. Success rates are even higher in newborn infants [19]. Nevertheless, there remains an important false negative reporting rate, which is difficult to quantify [20]. Given the operator-dependency, any doubt regarding coronary anomaly with potentially malignant course, should have further imaging [16]. Our study also shows that distal coronary tree imaging in children is superior with CMRA in comparison to echocardiography. This is another useful point of note from this study for clinical follow up of coronary disease in children. Multidetector CT angiography has excellent success rates in determining coronary origins and is becoming routinely used clinically to detect coronary stenosis [21, 22]. However, it does involve potentially harmful radiation use in young children.

Unfortunately, coronary distensibility in children has been largely neglected in the published literature [2, 3, 23]. Although an important recognized variable in adults, [24] this factor has been ignored when generating echocardiographic normal values for coronary dimensions in children [2, 3, 23]. The given reference study [3] was chosen specifically as the details regarding how measurements are to be taken are most precise. Even this study neglects to mention whether values are to be taken in systole or diastole. From clinical experience using echocardiography, it is usually difficult to get clear pictures for each segment in both phases and so it is understandable that this point is not labored. However, our data from the dual-phase CMRA measurements shows that dimensions do indeed vary according to phase. Future normal values for coronary dimensions in children should note this finding and provide phase-specific references.

## Conclusions

This study shows that echocardiographic reference values should not be applied to CMRA measurements of coronary dimensions. Looking forward, the development of CMRA-specific reference values would be helpful, although such normative data is difficult to acquire in children. Our study confirms the perception that CMRA remains extremely successful in the identification of coronary origins in children but echocardiography remains superior in small infants. Our study also shows that CMRA is superior to echocardiography in the imaging of the distal coronary tree of children. Finally, the study also demonstrates the need to establish heart phase-specific coronary reference ranges.
